# USING DIGITAL HEALTH TO REDUCE BURDEN IN OLDER ADULTS WITH CANCER UNDERGOING CHEMOTHERAPY: A SYSTEMATIC REVIEW

**DOI:** 10.1093/geroni/igae098.3923

**Published:** 2024-12-31

**Authors:** Hyebeen Sim, Jinhee Shin, Kennedy Diema Konlan

**Affiliations:** Kangbuk Samsung Hospital, Seoul, Republic of Korea; Woosuk university, Jeollabuk-do, Ch’ungch’ong-bukto, Republic of Korea; University of Health and Allied Sciences, Ho, Volta, Ghana

## Abstract

Digital health interventions have the potential to enhance health outcomes and support older adults with cancer in managing their conditions. However, the specific benefits and components of these interventions for this population remain inadequately defined. This systematic review aims to evaluate the effectiveness of digital health interventions in alleviating the burden of chemotherapy for older adults with cancer. This systematic review used PRISMA guidelines and the PICO framework. The Searches were conducted across five databases (CINAHL, Embase, PubMed, Cochrane Library, and Web of Science). Of the 5,683 articles screened, 13 were selected based on the inclusion criteria. Data extraction was performed by two authors using a predetermined matrix. The analysis was done using the interpretative thematic synthesis design. The digital health technologies examined included mobile apps, remote monitoring, and games, utilizing devices such as tablets, smartphones, and smartwatches. Significant benefits were observed for older adults with cancer undergoing chemotherapy, including improvements in symptom management, physical activity, nutritional support, and quality of life. Additionally, patients in the intervention groups experienced reductions in hospitalizations and treatment-related toxicities, along with improved efficacy in symptom monitoring. Digital health interventions show considerable promise in supporting older adults undergoing chemotherapy. However, further research is essential to clearly define the components of these digital health interventions for older adults with cancer and their specific impacts. Developing and adopting accessible digital health intervention applications that can be used at any time and place will be crucial in effectively preventing or alleviating the adverse effects of cancer treatment.

